# Cobalt and Nickel Stabilize Stem Cell Transcription Factor OCT4 through Modulating Its Sumoylation and Ubiquitination

**DOI:** 10.1371/journal.pone.0086620

**Published:** 2014-01-31

**Authors:** Yixin Yao, Yinghua Lu, Wen-chi Chen, Yongping Jiang, Tao Cheng, Yupo Ma, Lou Lu, Wei Dai

**Affiliations:** 1 Department of Environmental Medicine, New York University Langone Medical Center, Tuxedo, New York, United States of America; 2 Biopharmaceutical Research Center, Chinese Academy of Medical Sciences & Peking Union Medical College, Suzhou, China; 3 Institute of Hematology & Blood Disease Hospital, Chinese Academy of Medical Sciences & Peking Union Medical College, Tianjin, China; 4 Yupo Ma, Department of Pathology, The State University of New York at Stony Brook, Stony Brook, New York, United States of America; 5 Department of Medicine, David Geffen School of Medicine, University of California Los Angeles, Torrance, California, United States of America; 6 Department of Biochemistry and Molecular Pharmacology, New York University Langone Medical Center, Tuxedo, New York, United States of America; University of Kentucky, United States of America

## Abstract

Stem cell research can lead to the development of treatments for a wide range of ailments including diabetes, heart disease, aging, neurodegenerative diseases, spinal cord injury, and cancer. OCT4 is a master regulator of self-renewal of undifferentiated embryonic stem cells. OCT4 also plays a crucial role in reprogramming of somatic cells into induced pluripotent stem (iPS) cells. Given known *vivo* reproductive toxicity of cobalt and nickel metals, we examined the effect of these metals on expression of several stem cell factors in embryonic Tera-1 cells, as well as stem cells. Cobalt and nickel induced a concentration-dependent increase of OCT4 and HIF-1α, but not NANOG or KLF4. OCT4 induced by cobalt and nickel was due primarily to protein stabilization because MG132 stabilized OCT4 in cells treated with either metals and because neither nickel nor cobalt significantly modulated its steady-state mRNA level. OCT4 stabilization by cobalt and nickel was mediated largely through reactive oxygen species (ROS) as co-treatment with ascorbic acid abolished OCT4 increase. Moreover, nickel and cobalt treatment increased sumoylation and mono-ubiquitination of OCT4 and K123 was crucial for mediating these modifications. Combined, our observations suggest that nickel and cobalt may exert their reproductive toxicity through perturbing OCT4 activity in the stem cell compartment.

## Introduction

Cobalt [Co(II)] and Nickel [Ni(II)] are capable of crossing the placenta barrier and exerting their toxicity on the animal reproduction system, thus affecting embryonic development [1], [2]. Exposure of Ni(II) and Co(II) at a high concentration (100 µM) significantly reduced proliferation of inner cell mass and trophoblast cells [3]. The reduced proliferative ability of trophoblast cells compromises invasiveness of the embryo [3]. Intriguingly, exposure of Co(II) at a low concentration (1 µM) induces a highly organized inner cell mass with an abnormally large size [2]. Human exposure to cobalt and nickel occur environmentally and occupationally. It has been reported that there is a correlation between occupational exposure to nickel (refinery female workers) and delivery of newborns small-for-gestational-age [4]. Both soluble and insoluble nickel can potentially pose threat to human health. It has been reported that potential intracellular concentrations of nickel ion can reach the molar range after cell phagocytizes a crystalline NiS particle [5].

Octamer binding protein 4 (OCT4), SOX2, Krüppel-like factor 4 (KLF4), and MYC are important transcription factors that are capable of reprogramming somatic cells into pluripotent stem cells [6]–[8]. Induced pluripotent stem (iPS) cells possess the capacity of developing into an entire organism [9]. Hypoxia improves the rate of reprogramming differentiated cells into iPS cells [10]–[14]. Consistent with these findings, bovine blastocysts produced under a reduced oxygen tension exhibit significantly more inner cell mass (consisting of embryonic stem cells) than those maintained at a normal oxygen tension [15].

OCT4 is a stem cell transcription factor that activates or represses target gene expression depending on cellular context [16]–[18]. OCT4 and other stem cell factors including NANOG and SALL4 form a transcriptional network that controls pluripotency in ES cells [19]. *OCT4* mRNA and its protein are present in unfertilized oocytes; OCT4 protein is localized to pronuclei following fertilization [20]. *OCT4* mRNA levels drop dramatically after fertilization albeit OCT4 protein remains detectable in the nuclei of 2-cell embryos [20]. Zygotic *OCT4* expression is activated prior to the 8- cell stage, leading to the increase of both mRNA and protein [20].

OCT4 is subject to post translational modifications including phosphorylation [21]–[23], poly-ubiquitination [24], [25] and sumoylation [26]–[28]. For example, AKT1 phosphorylates OCT4 at threonine 235 (T235) in embryonic carcinoma cells [22]. The phosphorylation increases the stability of OCT4 and facilitates its nuclear localization and interaction with SOX2. OCT4 is also modified by sumoylation, which positively regulates its stability, chromatin binding, and transcriptional activity [26].

To understand whether toxicity of nickel and cobalt on embryonic development is partly mediated by their effect on stem cell transcription factors, we studied OCT4 expression in both primary stem cells and stem cell-derived cell lines treated with nickel or cobalt ions. We observed that Ni(II) and Co(II) significantly increased expression of OCT4 in a time- and concentration-dependent manner. Ni(II)- or Co(II)-induced OCT4 expression is primarily due to protein stabilization. Our further studies reveal that ROS produced as the result of Ni(II) and Co(II) exposure is responsible for OCT4 stabilization partly via modulating post-translational modifications.

## Results

### Ni(II) and Co(II) Induce OCT4

**Figure 1:**
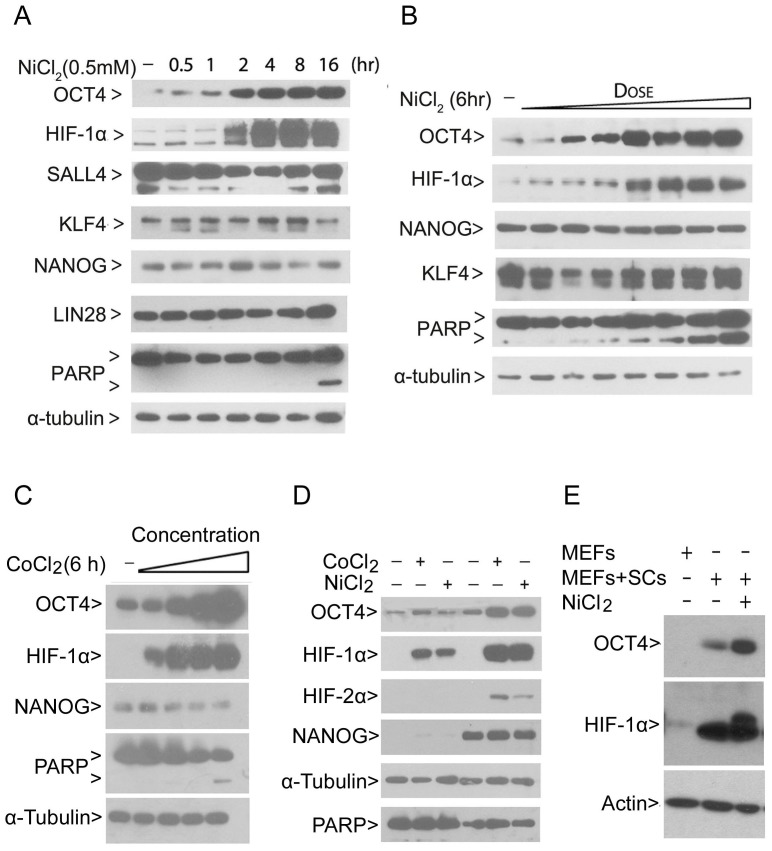
Induction of OCT4 expression by nickel and cobalt. A, Tera-1 cells treated with or without NiCl_2_ (0.5 mM) for various times were lysed and equal amounts of cell lysates were blotted with indicated antibodies. B, Tera-1 cells treated with various concentrations of nickel (e.g., 15.6, 31.3, 62.5, 125, 250, 500, and 1000 µM) were lysed and equal amounts of cell lysates were blotted with antibodies as indicated. C, Tera-1 cells were treated with CoCl_2_ at various concentrations (e.g., 125, 250, 500, and 1000 µM) and equal amounts of cell lysates were blotted with antibodies as indicated. D, Tera-1 and NT2/D1 cells were treated with NiCl_2_ or CoCl_2_ (0.5 mM) for 6 h and equal amounts of cell lysates were blotted with antibodies as indicated. E, Human embryonic stem cells (H1) grown on MEFs were treated with or without nickel (0.25 mM) for 24 h, after which cells were collected and lysed. MEFs cells grown separately without stem cells were also collected as control. Equal amounts of cell lysates were then blotted for OCT4.

To determine if expression of key stem cell transcription factors was affected by metal-induced stresses, Tera-1 cells (embryonic carcinoma origin) were treated with nickel chloride (NiCl_2_) for various times. Equal amounts of cell lysates were blotted with antibodies to a panel of transcription factors including OCT4, NANOG, KLF4, SALL4, and HIF-1α. As expected, HIF-1α levels were stabilized by Ni(II) (Fig. 1A and 1B) as the metal is known to be a hypoxic mimetic [29]. Interestingly, OCT4 protein levels, but not other key stem cell factors including SALL4, NANOG, and KLF4, also exhibited a time- and concentration-dependent increase (Fig. 1A and 1B). Cobalt, a metal with many overlapping properties with nickel, also induced the increase of OCT4, but not NANOG, in Tera-1 cells in a concentration-dependent manner (Fig. 1C). As expected, it induced HIF-1α as well given its known property as a hypoxic mimetic [29]. Ni(II) and Co(II) also induced OCT4 in NT2 cells (embryonic origin) although the magnitude of induction was not as great as seen in Tera-1 cells (Fig. 1D), suggesting that cell lines with different genetic backgrounds may respond to the metal stress differently. Supporting this, HIF-2α was not inducible in NT2 cells by either Ni(II) or Co(II) whereas it was induced in Tera-1 cells (Fig. 1D).

To further confirm that induction of OCT4 occurs in primary stem cells, we treated feeder-dependent human embryonic stem cells (H1, WiCell) with NiCl_2_. We observed that there is a basal level of OCT4 expression in H1 stem cells but not in feeder cells (murine embryonic fibroblasts). Nickel treatment significantly elevated the level of OCT4 (Fig. 1E). As expected, nickel induced expression of HIF-1α as well. In addition, we observed that nickel (or cobalt) treatment of human iPS cells could induce expression of OCT4 (data not shown). Moreover, chromium, another environmental metal toxicant, did not induce expression of OCT4 (data not shown). Combined, our observations are consistent with the notion that the steady-state level of OCT4 can be perturbed by exposure to nickel or cobalt ions.

### OCT4 Induction by Ni(II) or Co(II) was Not Due to Transcriptional Activation

**Figure 2:**
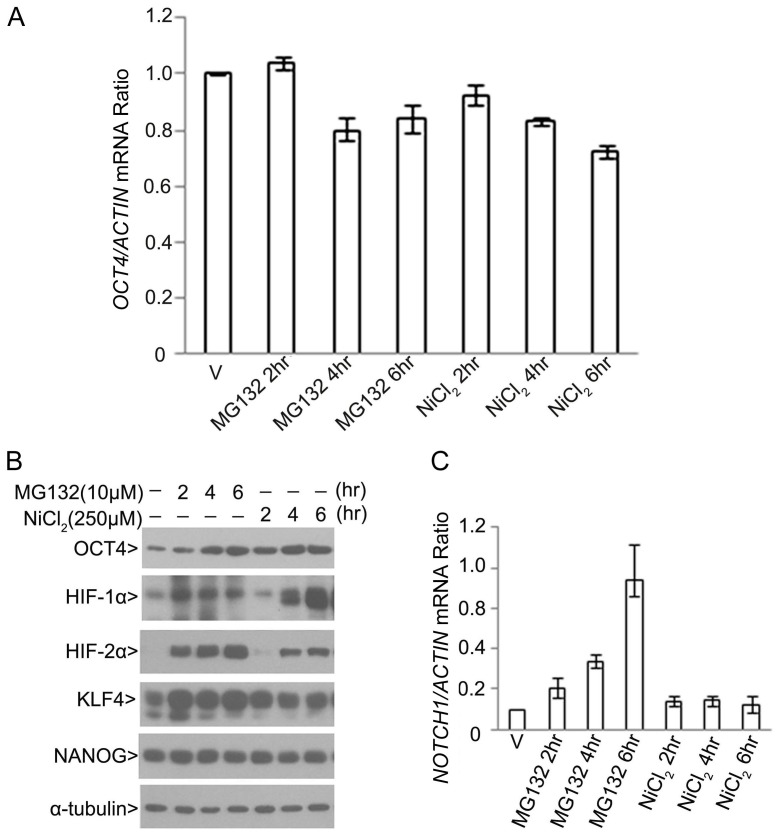
OCT4 induction by nickel or cobalt is not due to increased transcription. A, Tera-1 cells treated with MG132 (10 µM) or NiCl_2_ (0.25 mM) for various times were collected and total RNAs were extracted. OCT4 specific mRNA was measured using quantitative PCR and specific signals were normalized by β-actin mRNA levels. B, Tera-1 cells treated with MG132 or NiCl_2_ (0.25 mM) for various times were lysed and equal amounts of lysates were blotted with indicated antibodies. C, Quantification of expression of *NOTCH-1* mRNA in cells treated with MG132 or NiCl_2_ for various times.

To determine whether increased expression of OCT4 by Ni(II) or Co(II) was due to transcriptional activation, RNA samples extracted from Tera-1 cells treated with Ni(II) or MG132 were analyzed by quantitative polymerase chain reaction (qPCR). There was no increase in *OCT4* mRNA in cells treated with Ni(II) and/or MG132 whereas Ni(II) or MG132 greatly stimulated the accumulation of OCT4 and HIF-1α protein levels (Fig. 2A and 2B). As control, we analyzed *NOTCH1* mRNA levels via qPCR as it was under control of SALL4, also a stem cell transcription factor [30]. We observed that *NOTCH1* mRNA was significantly increased in cells treated with MG132 but not with Ni(II) (Fig. 2C).

### Cobalt and Nickel Prolong the Half-life of OCT4 in Tera-1 Cells

**Figure 3:**
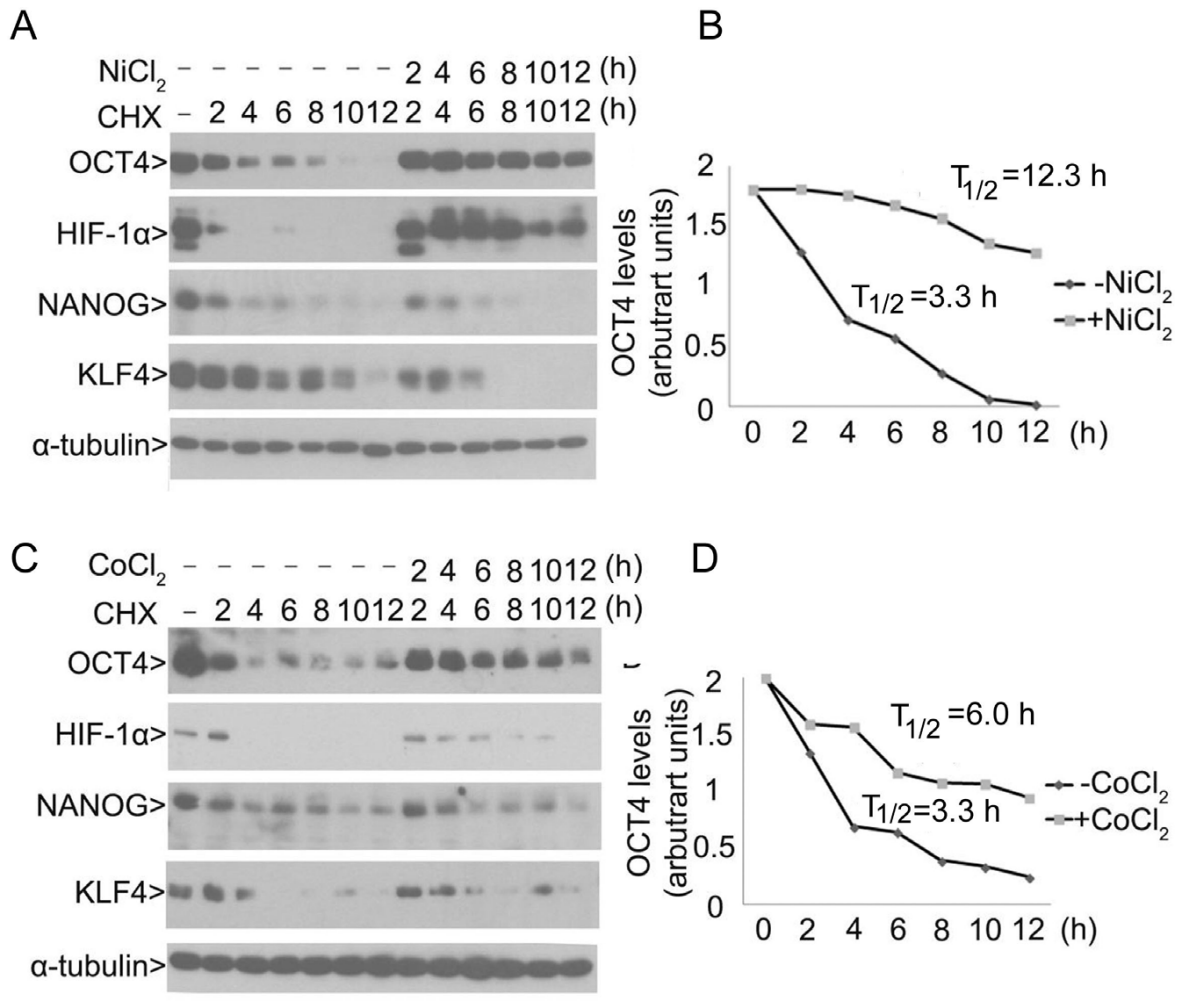
Cobalt and nickel prolong the half-life of OCT4. A. Tera-1 cells were treated with cycloheximide (CHX) in the presence of absence of NiCl_2_ for various times. Equal amounts of cell lysates were blotted with indicated antibodies. B. OCT4 signals shown in *A* were quantified and plotted. T_1/2_ denotes an estimated half-life. C. Tera-1 cells were treated with CHX in the presence of absence of CoCl_2_ for various times. Equal amounts of cell lysates were blotted with indicated antibodies. D. OCT4 signals shown in *C* were quantified and plotted. T_1/2_ denotes an estimated half-life.

To confirm that Ni(II) or Co(II) affects OCT4 protein stability, Tera-1 cells treated with cycloheximide (CHX), a chemical that blocks new protein synthesis, in the presence or the absence of Ni(II). At various times of treatment, cells were collected and equal amounts of cell lysates were blotted for OCT4, as well as other transcription factors. Ni(II) significantly stabilized the level of OCT4, but not NANOG and KLF4, in cells treated with CHX and prolonged its half-life (Fig. 3A and 3B). As expected, Ni(II) treatment also greatly stabilized HIF-1α. In addition, Co(II) significantly prolonged the half-life of both OCT4 and HIF-1α in cells treated with CHX (Fig. 3C and 3D). Combined, these studies indicate that OCT4 increase after Ni(II) or Co(II) treatment is primarily due to an increased protein stability.

### Post-translational Modifications of OCT4 are Enhanced by Co(II)

**Figure 4:**
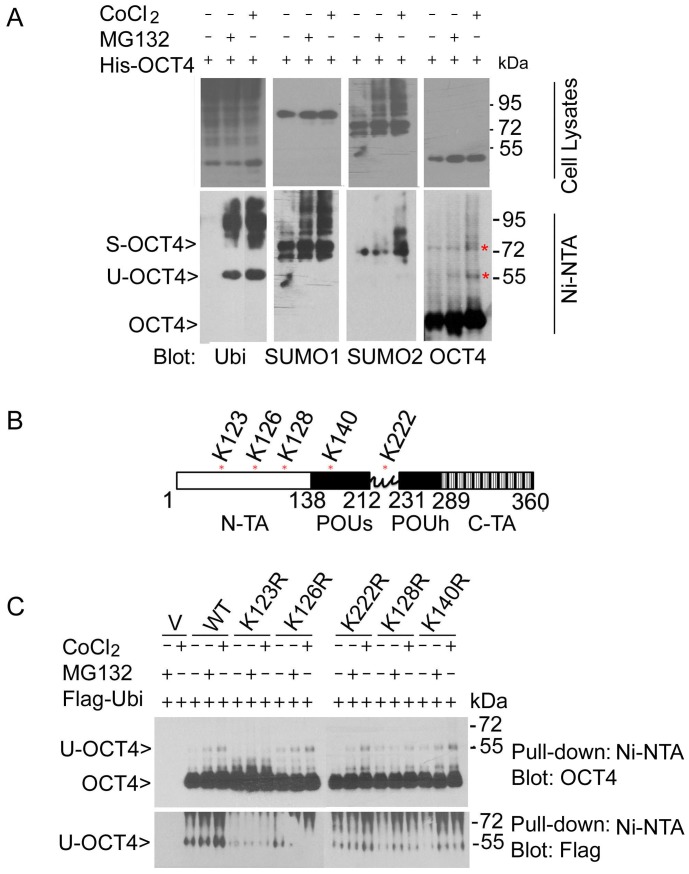
OCT4 is post-translationally modified. A. HEK293T cells transfected with a His_6_-OCT4 expression plasmid for 48 h were treated with vehicle, MG132 or CoCl_2_ for 3 h. Equal amounts of cell lysates were incubated with Ni-NTA resin. Proteins specifically bound to the resin, along with the lysate inputs, were blotted with antibodies to OCT4, SUMO-1, SUMO-2, and ubiquitin. S-OCT4 and U-OCT4 denote sumoylated and ubiquitinated OCT4, respectively. B. Schematic presentation of OCT4 domain, as well as lysine residues potential for ubiquitination. C. HEK293T cells were co-transfected with various OCT4 expression plasmids and a Flag-tagged ubiquitin expression plasmid for 48 h. After transfection, cell lysates of various treatments were incubated with Ni-NTA resin. Proteins specifically bound to the resin, along with total cell lysates, were blotted with antibodies to Flag and OCT4.

OCT4 protein stability is modulated by ubiquitination and sumoylation [26]–[28]. To test whether Co(II) or Ni(II) stabilizes OCT4 through affecting post translational modifications including ubiquitination and/or sumoylation, His_6_-OCT4 ectopically expressed in HEK293T cells was pulled down by Ni-NTA resin. We used ectopic expression system in HEK293 cells partly because endogenous OCT4 in Tera-1 migrated at or near 55 kDa position, which interfered with various biochemical studies (e.g., co-immunoprecipitation). Western blotting analysis showed that many slow mobility bands of OCT4 were detected in pull-down samples that these bands were induced/enhanced after treatment with Co(II) or MG132 (Fig. 4A). Moreover, major bands that were modified by SUMO-1 (∼75 kDa) and ubiquitin (55 kDa) co-migrated with slow mobility bands of OCT4 (marked by *), indicating that these bands are OCT4-specific. Although pull-down samples were also positive for SUMO-2 modification its level appeared to be much lower than that of SUMO-1 modification (Fig. 4A, SUMO-1 and SUMO-2 blots). Enhanced modifications of OCT4 were also demonstrated with cells treated with Ni(II) (data not shown).

### K123 is Important for Mono-sumoylation and Mono-ubiquitination of OCT4

To identify potential lysine residues that were modified by ubiquitination, we analyzed OCT4 amino acid sequences for optimal ubiquitination sites using the criteria available (www.ubpred.org). Four lysines sites (K123, K126, K128, and K140) with the highest scores along with a low score lysine site K222 were subjected to mutagenic analysis. The relative position of these sites to OCT4 domains is shown in Fig. 4B. K123 appeared to be critical for mediating mono-ubiquitination of OCT4 as its mutation into R largely abolished 55 kDa band (Fig. 4C, Upper panel). Neither Co(II) nor MG132 induced ubiquitin-modified OCT4 in K123 mutant. Blotting with antibody to Flag (part of ectopic ubiquitin) confirmed the importance of K123 in mediating ubiquitination of OCT4 although other mutants appeared to have a negative effect on OCT4 sumoylation (Fig. 4C, Lower panel).

**Figure 5:**
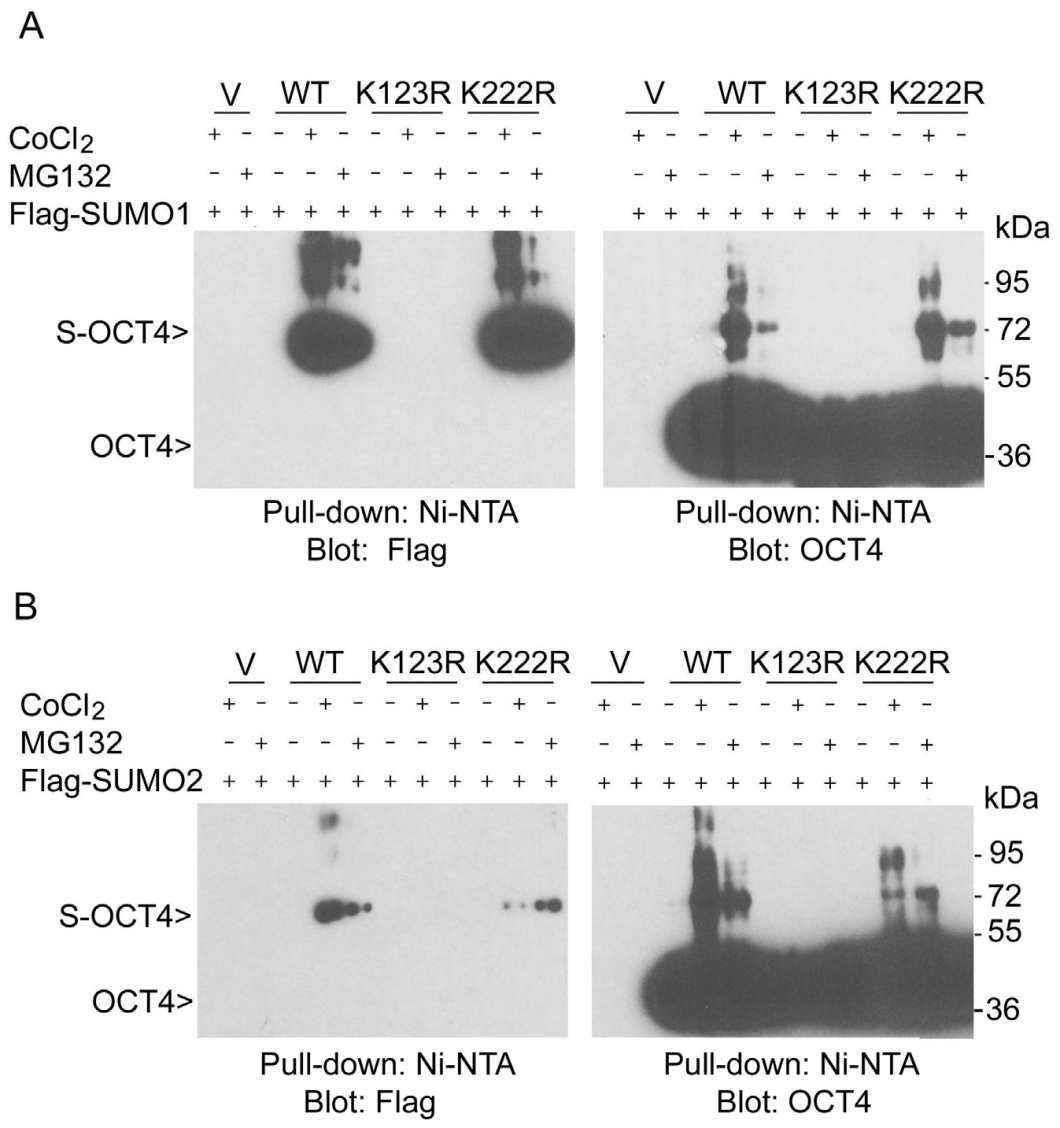
Cobalt enhances sumoylation of OCT4 at K123. A. HEK293T cells were co-transfected with various OCT4 expression plasmids and a FLAG-tagged SUMO-1 expression plasmid for 48 h followed by treatment with CoCl_2_ or MG132 for 3 h. Cell lysates of various treatments were incubated with Ni-NTA resin. Proteins specifically bound to the resin, along with total cell lysates, were blotted with antibodies to Flag and OCT4. B. HEK293T cells were co-transfected with various OCT4 expression plasmids and a FLAG-tagged SUMO-2 expression plasmid for 48 h followed by treatment with CoCl_2_ or MG132 for 3 h. Cell lysates of various treatments were incubated with Ni-NTA resin. Proteins specifically bound to the resin, along with total cell lysates, were blotted with antibodies to Flag and OCT4.

K123 but not K222 of OCT4 is modified by sumoylation [26], [27]. To identify potential site(s) whose SUMO-modification can be affected by Ni(II) or Co(II) treatment, we co-transfected HEK293 cells with Flag-tagged *SUMO-1* (or *SUMO-2*) and OCT4 (or its mutant) expression constructs. Pull-down analysis coupled with immunoblotting confirmed that K123 was indeed a site that could be modified by SUMO-1 and SUMO-2 (Fig. 5A and 5B). SUMO modification was greatly enhanced/induced after Co(II) and MG132 treatment. Blotting with the antibody against the FLAG tag confirmed that modification by SUMO-1 was much more pronounced than that by SUMO-2 (Fig. 5A and 5B), which is consistent with the early observation (Fig. 4A).

### K123 is Important for OCT4 to Bind to Chromatin after Co(II) Exposure

**Figure 6:**
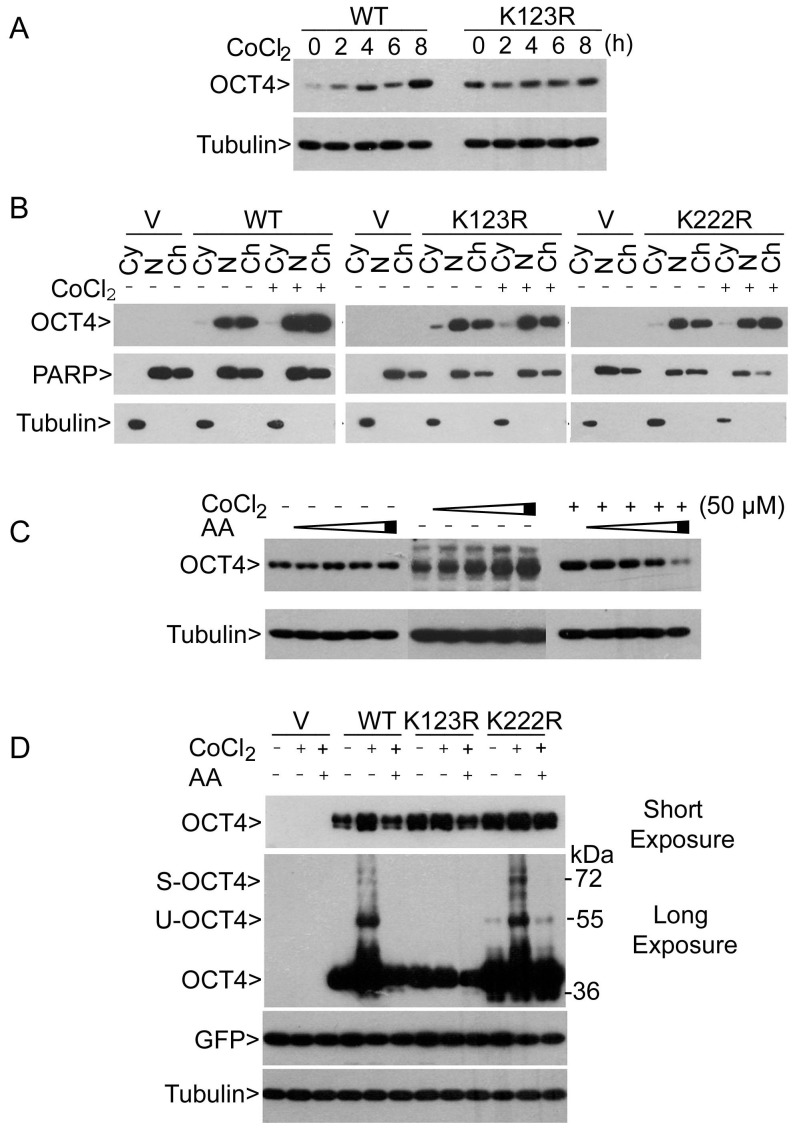
K132 is important for OCT4 chromatin association after CoCl_2_ treatment. A. HEK293 cells were transfected with WT OCT4 or OCT4^K123R^ for 48 h and then treated with CoCl_2_ for various times. Equal amounts of cell lysates were then blotted for OCT4 and α-tubulin. B. HEK293 cells were transfected with WT OCT4 or various mutant constructs for 48 h followed by treatment with CoCl_2_ for 3 h. Cells with various treatments were lysed and separated into cytoplasmic (Cy), soluble nuclear (N) and chromatin (Ch) fractions. Equal amounts of proteins from each fraction were blotted with antibodies to OCT4 and α-tubulin. C. HEK293 cells transfected with WT OCT4 for 48 h were treated with CoCl_2_ (0, 50, 100, 150, and 200 µM) and/or ascorbic acid (AA) (0, 50, 100, 150, and 200 µM) for 3 h. At the end of treatments, cells were lysed and equal amounts of proteins were blotted for OCT4 and α-tubulin. D. HEK293 cells were transfected with WT OCT4 or various mutant constructs for 48 h followed by treatment with CoCl_2_ or ascorbic acid (AA) for 3 h. GFP expression plasmid was used for co-transfection to monitor transfection efficiency. Cell lysates of various treatments were blotted with antibodies to OCT4, GFP and α-tubulin. Both short and long exposures of OCT4 blots were shown. S-OCT4 and U-OCT4 denote sumoylated and ubiquitinated OCT4, respectively.

OCT4 functions are primarily mediated through binding to promoters of target genes, thereby regulating their expression [31]. To determine whether OCT4 modifications on K123 were important for its induction by Co(II), HEK293 cells were transfected with a wild-type (WT) construct of OCT4 or its mutant OCT4^K123R^ and treated with Co(II) for various times, after which cell lysates were blotted for OCT4. In contrast to WT OCT4, OCT4^K123R^ expression was not induced by Co(II) (Fig. 6A). We then asked whether K123 mutation affected its subcellular localization. Immunoblot analysis of fractionated cell lysates revealed that both WT OCT4 and OCT4^K123R^ were associated with chromatin in untreated cells; however, Co(II) exposure stimulated the increase of WT OCT4 but not OCT4^K123R^ on chromatin (Fig. 6B). Intriguingly, OCT4^K222R^ remained elevated after Co(II) treatment, which behaved in a manner similar to that of wild-type OCT4.

As both Co(II) and Ni(II) are capable of generating reactive oxygen species (ROS) [29] , we asked whether induction of OCT4 by these metal was partly mediated through ROS. Tera-1 cells treated with Co(II) were supplemented with various concentrations of ascorbic acid (AA) that inhibits ROS production. Co(II) was used as it is a stronger ROS inducer than Ni(II) [29]. Whereas AA alone did not significantly modulate OCT4 levels it repressed Co(II)-induced OCT4 (Fig. 6C). Significantly, in combination with Co(II), AA destabilized the steady-state level of OCT4 in a concentration-dependent manner. Our further analysis revealed that AA treatment also reduced OCT4 modifications (sumoylation and monoubiquitination) mediated by K123 (Fig. 6D).

### Co(II) Increases OCT4 Activity by Modulating SUMO-1-modification on K123

**Figure 7:**
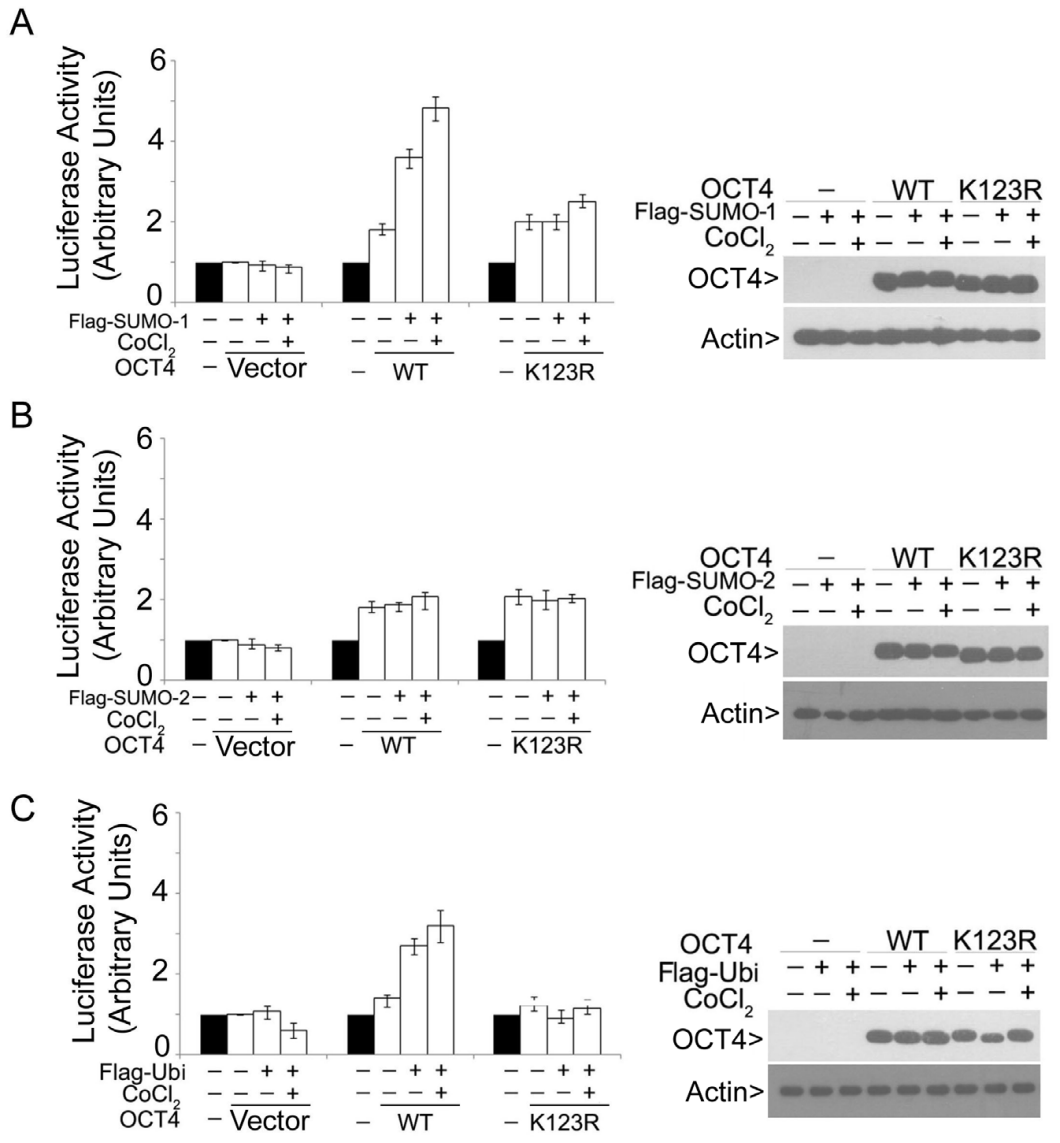
Cobalt stimulates SUMO-1-mediated transcriptional activity of OCT4. A. HEK293T cells were seeded in triplicate and co-transfected for 42 h with 6W-37tk-luc reporter, Flag-SUMO-1 (or Flag-SUMO-2 or Flag-ubiquitin), and WT OCT4 (or OCT4^K123R^) expression plasmids. Transfected cells were then treated with CoCl_2_ (50 µM) for 6 h, after which cells were lysed. Equal amounts of cell lysates were assayed for firefly luciferase activities as described in Materials and Methods. Data are expressed as fold-changes after normalization by the renilla luciferase activity. Each experiment was repeated for at least three times. Samples from each transfection were also blotted with antibodies to OCT4 and α-tubulin.

To determine whether K123 was important for transcriptional functions of OCT4, we co-transfected HEK293T cells with an OCT4 [or mutant (OCT4^K123R^) expression construct and a luciferase reproter construct driven by a thymidine kinase promoter fused to six copies of octamer (OCT4 monomer binding sequence]. Flag-tagged-SUMO-1, SUMO-2, or ubiquitin expression construct was also used for co-transfection. After transfection for 48 h, equal amounts of cell lysates were analyzed for luciferase activities. Ectopical expression of SUMO-1 greatly enhanced reporter gene activities in cells expressing WT OCT4 (Fig. 7A), which was further boosted by Co(II) treatment. However, the reporter gene activities were not significantly increased when cells were co-transfected with constructs expressing SUMO-1 and mutant OCT4 in the presence or absence of Co(II). Moreover, co-transfection with plasmid constructs expressing SUMO-2 and OCT4 (WT or mutant) did not significantly modulate the reporter gene activities (Fig. 7B), suggesting that SUMO-2 may not be used as a major modification *in vivo* and/or that SUMO-2 modification does not significantly affect OCT4 activity. Interestingly, co-expression of ubiquitin and WT-OCT4, but not OCT4^K123R^, significantly boosted the reporter gene activities although Co(II) treatment did not significantly increase the activity (Fig. 7C). Expression of various OCT4 constructs was monitored by immunoblotting (Fig. 7, right panels).

## Discussion

OCT4 is a master regulator of proliferation and self-renewal of embryonic stem cells [6], [32]. *OCT4* mRNA and protein are present in unfertilized oocytes, acting as an important maternal factor to regulate embryonic development [20]. The inner cell mass and trophoblast layer regulated by OCT4 are crucial because both contribute to the normal development of healthy embryos [33]. Given its importance, OCT4 expression is tightly controlled and any perturbations of its expression are expected to have an adverse effect on cell proliferation and differentiation [32].

Nickel and cobalt are both belong to group VII in the periodic chart, thus having similar chemical properties. Cobalt also shares similar features with nickel on iron regulation [34]. An earlier *in vivo* study showed Ni(II) reduced mouse embryo implantation frequency significantly when it was injected to mice during the pre-implantation stage [35]. The size and weight of mouse litters were reduced in the treated groups as compared with that of control group. In a separate study, it has been shown that Ni(II) treated mice exhibit a high rate of embryo resorption, abnormal fetuses, and stillborn [36]. Nickel exposure also causes a significant reduction in the trophoblast area and inner cell mass [2]. Reduced proliferative ability of trophoblast cells appears to be associated with compromised invasiveness of the embryo [3]. Our current studies strongly suggest that embryonic toxicity caused by nickel or cobalt exposure is likely due, at least impart, to altered expression and activity of OCT4.

It has been shown that nickel and cobalt toxicity and carcinogenicity are mediated through ROS production [29]. Using the electron paramagnetic resonance spin trapping approach, Hanna et al. have shown that various Co(II) complexes generate ROS from the reaction of hydrogen peroxide under physiological conditions [37]. Moreover, it has been suggested that depletion of glutathione may be a possible mechanism of oxidative stress induced by nickel [38], [39]. Many stem cell transcription factors function as onco-proteins, thus promoting cell proliferation and facilitating malignant transformation when their expression and activities are deregulated [40]–[43]. Given that OCT4 controls expression of many transcription factors including NANOG, SALL4, Myc and SOX2 [19], [44], it is tempting to speculate that Co(II) or Ni(II) carcinogenesis in the stem cell compartment may be partly due to an enhanced activities of OCT4 and its downstream targets.

OCT4 has two distinct DNA binding domains, POU domain (a.a.138–212 in human) and homeobox (a.a. 231-189) which independently bind half-sites of the canonical octamer motif [45]. This flexibility allows OCT4 to form heterodimers with other transcription factors, as well as to form homodimers [46]. Post-translational modifications are known to impact on protein conformation. In fact, it has been shown that OCT4 protein stability and transcriptional activities are subjected to the regulation by post-translational modifications including phosphoylation [21], [22], sumoylation [26]–[28] and poly-ubiquitination [24], [25]. Here we showed that OCT4 exhibits multiple modifications including ubiquitination and sumoylation, levels of which appear to correlated with OCT4 stability. Moreover, modifications of OCT4 can be induced by exposure to Co(II) or Ni(II). We have observed that OCT4 can be modified by SUMO-1 and SUMO-2. We have also demonstrated that Ni(II) and Co(II) enhance SUMO-modification of OCT4, leading to its stabilization. These observations are consistent with early reports that SUMO-1-modification of OCT4 affects its stability, as well as its transcriptional activity [26], [27]. In this study, we also showed that OCT4 can be modified by SUMO-2 albeit its level appears to be lower than that of SUMO-1. Our luciferase assays suggest that SUMO-2 modification does not seem to be important for OCT4 transcriptional activities.

## Experimental Procedures

### Cell Lines and Antibodies

HEK293T, TERA-1 and NT2/D1 cell lines were obtained from the American Type Culture Collection (ATCC, Manassas, VA). Anti-HIF-1α antibody was purchased from Bethyl Laboratories (Montgomery, TX). Antibodies to α-tubilin, PARP, and HIF-2α were purchased from Cell Signaling Technology (Danvers, MA). Antibodies against GFP, NANOG, and OCT4 were purchased from Santa Cruz Biotechnology (Santa Cruz, CA). SALL4 antibody was purchased from Abcam (Cambridge, MA). Human embryonic stem cells (H1, WiCell, WI) were cultured using a feeder-dependent culture condition. These cells were maintained in DMEM-F12 (Invitrogen, USA) medium which was supplemented with 20% KSR, 10 ng/mL bFGF, 2mM GlutaMAX™-I, 0.1 mM MEM Non-Essential Amino Acids Solution, 1×β-mercaptoethanol. Cells were passed every other day after trypsinization. Mitomycin C treated murine embryonic fibroblasts (MEFs) were prepared as feeder cells.

### Site-directed Mutagenesis

Mutant OCT4 with lysine 123 (K123), K126, K128, K140, and/or K222 replaced with arginine (R) were carried out using the QuikChange Lightning Site-directed Mutagenesis kit from Stratagene (Santa Clara, CA) according to the instruction provided by the supplier. Individual mutations were confirmed by DNA sequencing service from Seqwright (Houston, TX). Plasmid transfection was carried out using Lipofectamine reagents from Life Technology according to manufacturer’s instruction.

### RNA Isolation and qPCR

Total RNA was isolated from cells with various treatments using TriZol reagent (Life Technology) and converted into cDNA using SuperScript III First-Strand Synthesis Supermix for qRT-PCR (Life technology). Briefly, cells were immediately lysed in the TRIZOL reagent. RNA (1 µg) was reverse transcribed using oligo dT by reverse transcriptase. The synthesized cDNA was then used for quantitative real-time PCR (qPCR), which was carried out using ABI 7300 Real-Time PCR System (Life Technology). Eexpression levels of various genes were normalized to the levels of *ACT-B* mRNA, and expressed as fold induction relative to the untreated control.

### Immunoprecipitation and Pulldown Assays

For OCT4 pulldown assay, HEK293T cells overexpressed with various OCT4 constructs were lysed in 8 M urea buffer (100 mM NaH_2_PO_4;_ 10 mM Tris-HCl ,pH 8.0; 8 M urea). After extensive washing, proteins on the resin were eluted in the SDS sample buffer and subjected to analysis by SDS-PAGE followed by Western blotting with appropriate antibodies.

### Cell Fractionation

Cytoplasmic, nuclear and chromatin fractions were obtained using a modification of the procedure of Jin and Felsenfeld [47]. Cells were washed 3 times with PBS, suspended in the hypotonic buffer (10 mM Tris-HCl, pH 7.4; 10 mM KCl; 1.5 mM MgCl_2_; and 1 mM Dithiothreitol) supplemented with inhibitor cocktails (10 mM Na-butyrate, 0.5 µg/mL aprotinin, 0.5 µg/mL leupeptin, and 1 µg/mL aprotinin). Cells were disrupted using a 25 gauge needle. Nuclei were pelleted and resuspended in a low salt buffer (20 mM Tris-HCl ,pH 7.4; 20 mM KCl, 1.5 mM MgCl_2_; 25% glycerol and 1 mM DTT). Nuclei were homogenized with a 25 gauge needle followed by the addition of an equal volume of a high salt buffer (20 mM Tris-HCl, pH 7.4; 1.2 M KCl; 1.5 mM MgCl_2_; 25% glycerol 0.2 mM EDTA and 1 mM DTT). Soluble nuclear fraction and insoluble materials were seperated by centrifugation (14000 g×15 min) at 4°C. Pellets were resuspended in Tris saline magnesium buffer (20 mM Tris-HCl, ,pH, 7.4; 150 mM NaCl; 2 mM CaCl_2_; 2 mM MgCl_2_). The resuspended nuclei were digested with 120 U/µL micrococcal nuclease (Fisher) for 12 min at 37°C. The reaction was stopped by adding EDTA (pH 8.0) to a final concentration of 10 mM. After centrifugation (2500 rpm×5 min), the supernatant S1 was collected. After passing four times through a 20-gauge needle followed by four passes through a 25-gauge needle, the pellets were resuspended in the lysis buffer plus with 0.25 mM EDTA and incubated on ice for 15 min followed by centrifugation (10,000 rpm×10 min). The supernatant S2 was then collected and combined with S1 as the chromatin binding fraction.

### Half-life Study

After transfection of HEK293T cells with either wild-type or various mutant His_6_-OCT4 expression plasmid constructs for 24 h, cycloheximide (CHX) was added at a final concentration of 50 µg/ml to block new protein synthesis. Cells were harvested at various times post CHX treatment (2, 4, 6, 8, 10, and 12 h). Equal amounts of cell lysates were blotted for OCT4.

### Luciferase Reporter Gene Assays

Plasmid construct expressing firefly (Photinus pyralis) luciferase gene driven by the *OCT4* promoter was kindly provided by Dr. Yupo Ma (SUNY Stony Brook). Additional plasmid constructs (6XW, PORE, and MORE reporters) were gifts from Dr. Michael Atchison (University of Pennsylvania). HEK293T cells seeded in 12-well plate for 16 h. The total amount of DNA per well was equalized to 1.6 µg with carrier plasmid. Cells were co-transfected with the firefly reporter plasmid (0.3 µg/well), Flag tagged ubiquitin (or SUMO-1 and SUMO-2) plasmid (0.6 µg/well) RL Renilla luciferase reporter plasmid (Promega, Madison, WI; for monitoring transfection efficiency), and an *OCT4* expression plasmid (0.6 µg). Cells were then lysed and luciferase activities were measured using the Dual-Luciferase Reporter Assay System (Promega). Cell lysates were also blotted with antibodies to OCT4.

### Statistical Analysis

Data were represented as the mean ± SD. Differences between mean values of various samples were compared by Statistical Package for the Social Sciences (SPSS) software by two tailed Student *t* test. The differences were considered significant at P value ≤ 0.05.
